# Successful management by laparoscopic total gastrectomy following embolization for gastric cancer with overt bleeding: two case reports

**DOI:** 10.1093/jscr/rjaf211

**Published:** 2025-04-08

**Authors:** Ayaka Shibano, Akihiro Cho, Toshihiko Mouri, Yukiko Niwa, Takeshi Ishita, Moe Tanemura, Toshiya Sugishita, Ryota Higuchi, Masaho Ota, Satoshi Katagiri

**Affiliations:** Division of Gastroenterological Surgery, Tokyo Women’s Medical University, Yachiyo Medical Center, 477-96 Owadashinden, Yachiyo, Chiba, Japan; Division of Gastroenterological Surgery, Tokyo Women’s Medical University, Yachiyo Medical Center, 477-96 Owadashinden, Yachiyo, Chiba, Japan; Division of Gastroenterological Surgery, Tokyo Women’s Medical University, Yachiyo Medical Center, 477-96 Owadashinden, Yachiyo, Chiba, Japan; Division of Gastroenterological Surgery, Tokyo Women’s Medical University, Yachiyo Medical Center, 477-96 Owadashinden, Yachiyo, Chiba, Japan; Division of Gastroenterological Surgery, Tokyo Women’s Medical University, Yachiyo Medical Center, 477-96 Owadashinden, Yachiyo, Chiba, Japan; Division of Gastroenterological Surgery, Tokyo Women’s Medical University, Yachiyo Medical Center, 477-96 Owadashinden, Yachiyo, Chiba, Japan; Division of Gastroenterological Surgery, Tokyo Women’s Medical University, Yachiyo Medical Center, 477-96 Owadashinden, Yachiyo, Chiba, Japan; Division of Gastroenterological Surgery, Tokyo Women’s Medical University, Yachiyo Medical Center, 477-96 Owadashinden, Yachiyo, Chiba, Japan; Division of Gastroenterological Surgery, Tokyo Women’s Medical University, Yachiyo Medical Center, 477-96 Owadashinden, Yachiyo, Chiba, Japan; Division of Gastroenterological Surgery, Tokyo Women’s Medical University, Yachiyo Medical Center, 477-96 Owadashinden, Yachiyo, Chiba, Japan

**Keywords:** gastric cancer, haemorrhage, laparoscopic total gastrectomy, preoperative embolization

## Abstract

Gastric cancer (GC) bleeding is typically silent and chronic. However, active bleeding from GC can lead to life-threatening conditions. Endoscopic haemostasis is sometimes difficult and emergency surgery is associated with high postoperative complication rates. We encountered two cases of life-threatening GC haemorrhage. The patients were successfully treated with transcatheter arterial embolization and subsequent elective laparoscopic total gastrectomy. Preoperative embolization is effective for subsequent minimally invasive elective surgery in patients with severe bleeding from GC.

## Introduction

Although gastric cancer (GC) bleeding is usually silent and chronic, unlike peptic ulcer bleeding, active bleeding may cause hypovolemic shock and life-threatening conditions. Therefore, prompt haemostasis is crucial for improving clinical outcomes [1]. Endoscopic haemostasis is regarded as the first-line treatment. However, gastroenterologists sometimes encounter endoscopic haemostasis failure [2, 3]. Although radical gastrectomy can be an ideal treatment approach for both curing GC and controlling bleeding [4], emergency gastrectomy is associated with high morbidity and mortality [5, 6]. Herein, we present two cases of life-threatening haemorrhages that were successfully treated with transcatheter arterial embolization (TAE) and subsequent elective laparoscopic total gastrectomy.

## Case presentation

### Patient 1

**Figure 1:**
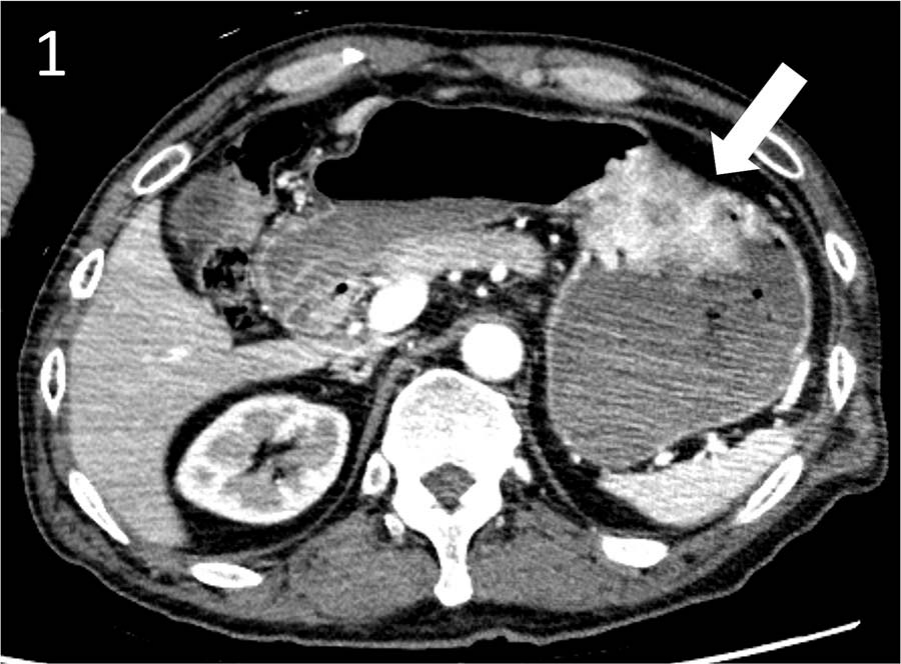
Contrast-enhanced computed tomography image showing a gastric hyper vascular mass on the lesser curvature (arrow).

**Figure 2:**
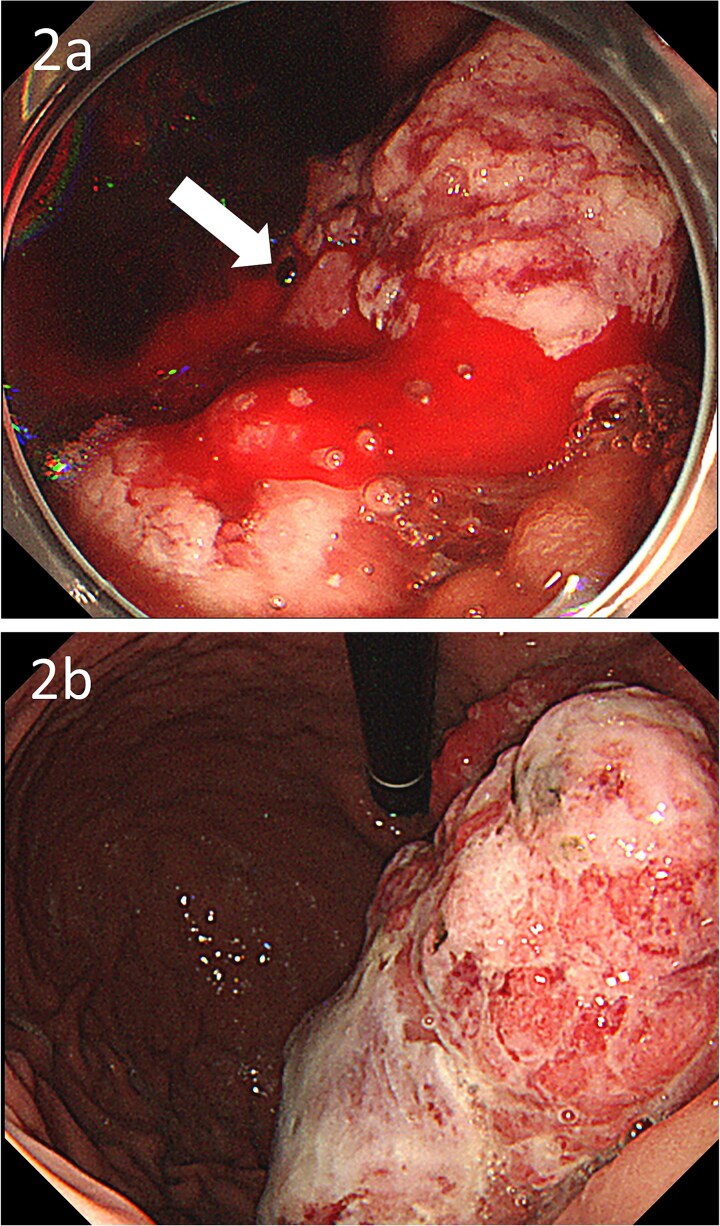
Gastroscopic images; (a) endoscopy showing a large type 1 oozing tumour (arrow); (b) endoscopy after embolization showing no bleeding.

**Figure 3:**
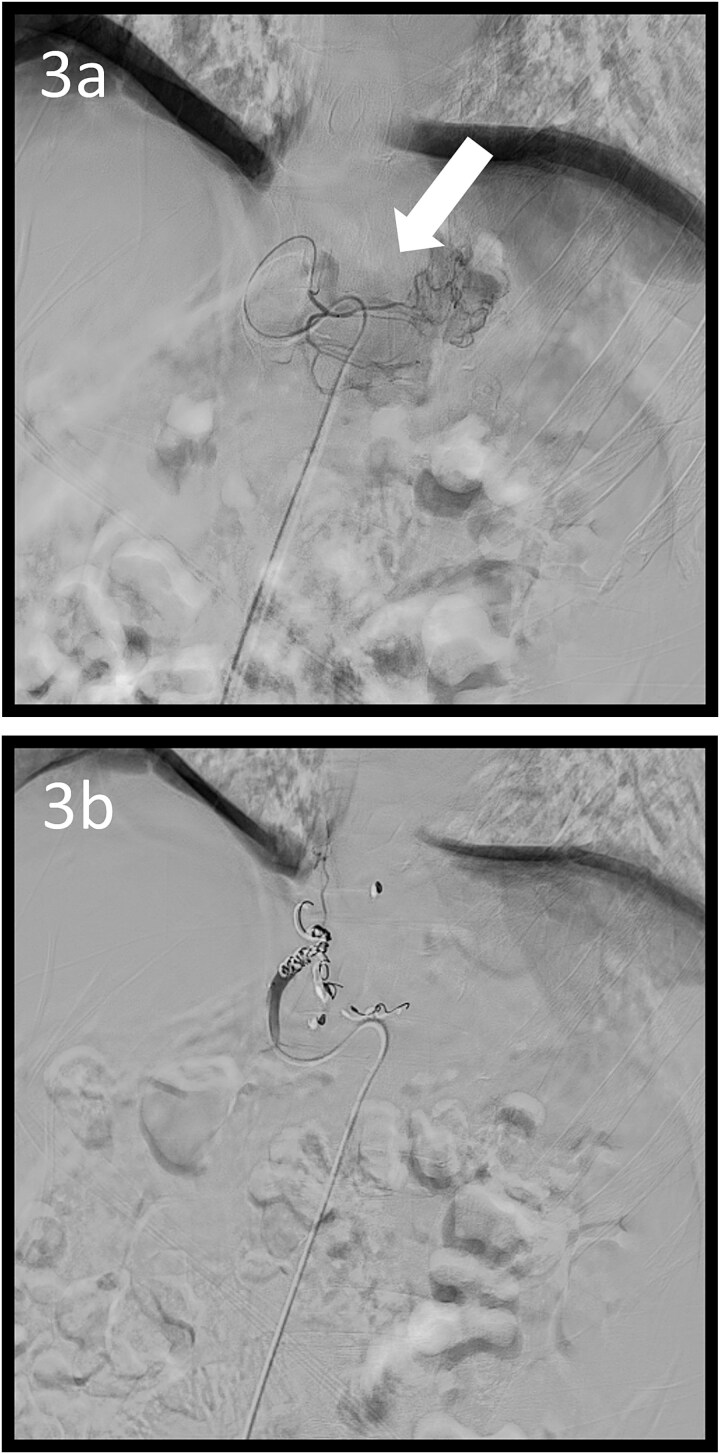
Angiographic images; (a) the left gastric arteriography showing tumour staining (arrow); (b) the left gastric artery was coil embolized.

A 59-year-old man presented to the emergency department with loss of consciousness and hematemesis. His blood pressure was 90/50 mmHg. The patient’s haemoglobin level was 7.9 g/dL. The patient was haemodynamically unstable, and 12 units of red blood cell (RBC) concentrate were administered. Contrast-enhanced computed tomography (CT) revealed a gastric hyper-vascular mass on the lesser curvature (Fig. 1). Emergency upper gastrointestinal (GI) endoscopy performed under endotracheal intubation and ventilatory management revealed a large type 1 oozing tumour (Fig. 2a). Haemostatic procedures were not performed due to technical difficulties. Emergency angiography revealed a tumour stain from the left gastric artery, which was coil embolized (Fig. 3). Upper GI endoscopy 2 days later revealed no bleeding (Fig. 2b). After embolization, the patient became haemodynamically stable. It was later discovered that he had undergone open omental filling at another hospital 1 year prior due to GC perforation and left his disease untreated for a year at his own discretion. The patient received combination chemotherapy with S-1 and oxaliplatin, which was remarkably effective. Eight months after embolization, laparoscopic total gastrectomy with Roux-en-Y reconstruction and lymph node dissection were performed. The final histopathological diagnosis was T2N0M0 stage IB. Macroscopic findings revealed a type 1 tumour in the upper part of the stomach. Histopathological findings revealed that the tumour was a well-differentiated tubular adenocarcinoma. All surgical margins were negative. The patient had an uneventful postoperative course and was discharged 7 days after surgery. The patient was well, with no recurrence noted during the 35 months of follow-up.

### Patient 2

**Figure 4:**
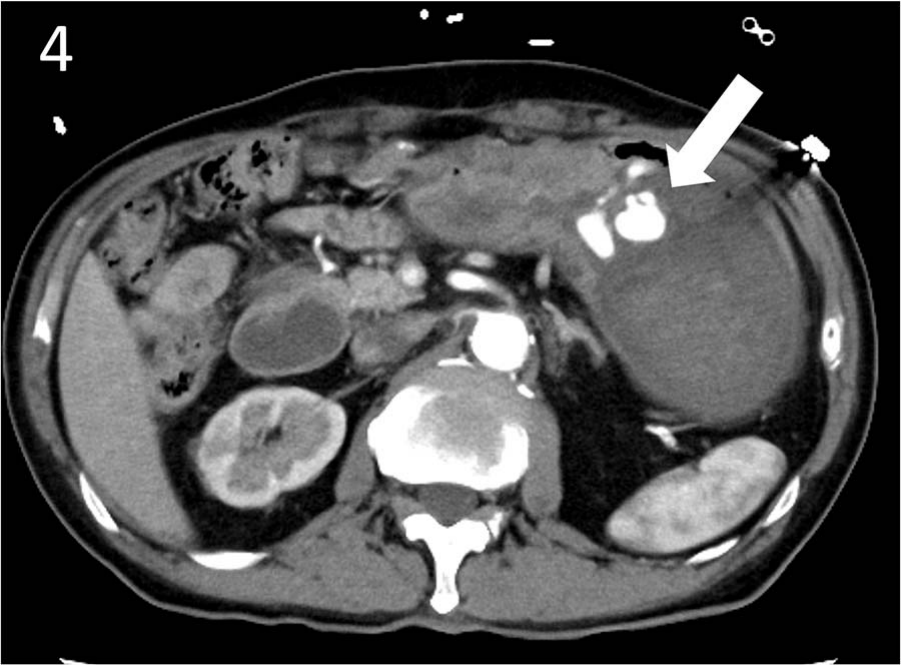
Contrast-enhanced computed tomography image showing active contrast extravasation in the stomach (arrow).

**Figure 5:**
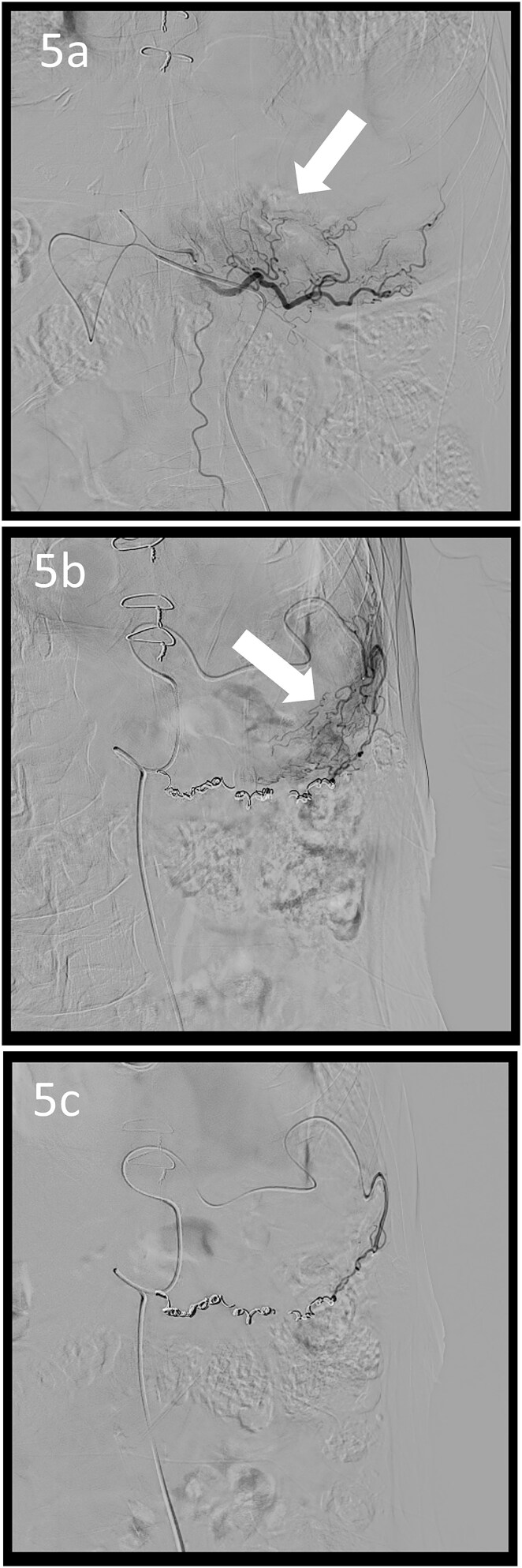
Angiographic images; the right (a) and left (b) gastroepiploic arteriograms showing tumour staining (arrows); (c) both arteries were coil embolized.

**Figure 6:**
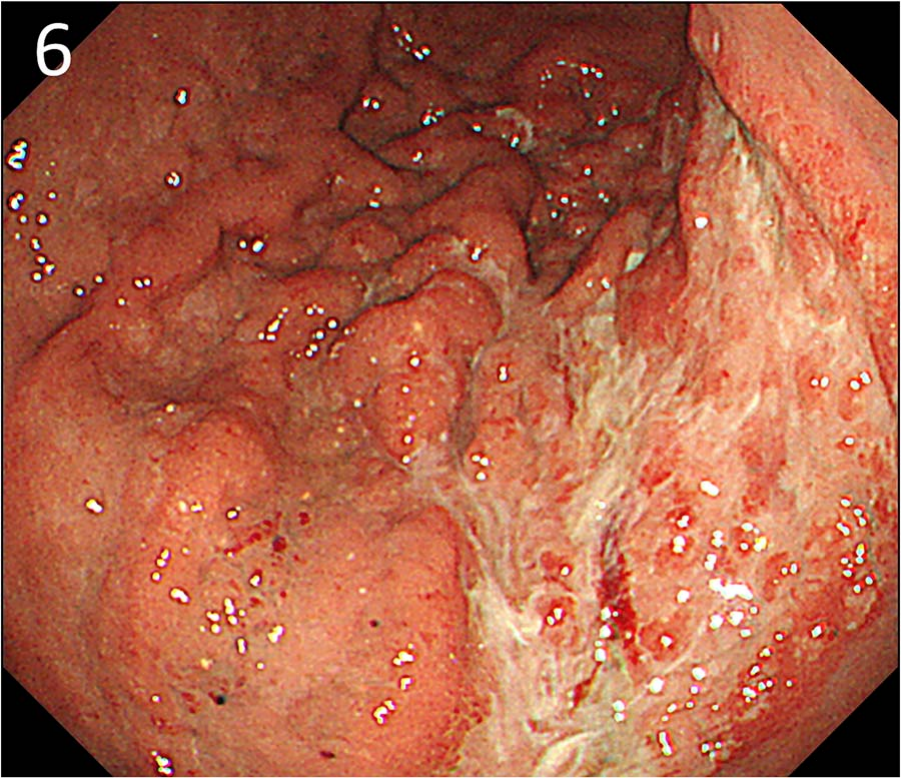
Endoscopy after embolization showing a type 4 tumour without bleeding.

A 78-year-old man was transferred to our hospital because of hypovolemic shock. He had already received 10 units of RBC concentrate. His blood pressure was 97/64 mmHg. The patient’s haemoglobin level was 8.2 g/dL. Contrast-enhanced CT showed active contrast extravasation in the stomach (Fig. 4). Emergency angiography revealed tumour staining in both the left and right gastroepiploic arteries, which were coil embolized (Fig. 5). Upper GI endoscopy 2 days later revealed a type 4 tumour without bleeding (Fig. 6). The patient was diagnosed with resectable GC, and underwent laparoscopic total gastrectomy with Roux-en-Y reconstruction and lymph node dissection. The final histopathological diagnosis was T4aN3aM0 stage IIIC. Macroscopic findings revealed a type 4 tumour of the stomach, and histopathological findings revealed that the tumour was a poorly differentiated adenocarcinoma with squamous cell differentiation. All surgical margins were negative. The patient developed a minor leakage that resolved spontaneously. The patient has remained recurrence-free for 12 months.

## Discussion

Failed management of GC bleeding is associated with poor prognosis [1]. The American, European, and Japanese Guidelines for the diagnosis and management of non-variceal upper GI bleeding recommend early upper GI endoscopy and endoscopic therapy following prompt hemodynamic resuscitation [7–9]. However, gastroenterologists sometimes encounter endoscopic haemostasis failure in cases of GC bleeding [2, 3]. The technical success rate of endoscopic haemostasis for GC bleeding ranges from 83% to 100% [2, 10, 11]. The mean interval between initial haemostasis and recurrent bleeding is 6 days, and the rebleeding rate after initial endoscopic haemostasis ranges from 28.3% to 49% [10, 11]. The presence of large bleeding lesions (>2 cm) and non-exposed vessel bleeding with a tumour are significant predictive factors for endoscopic haemostasis failure [2]. Although radical gastrectomy can be an ideal treatment approach for both curing GC and controlling bleeding [4], surgeons face many challenges, including haemodynamic instability, insufficient oncologic work-up, and emergent surgery which are associated with a low curative resection rate and high rates of mortality and morbidity [5, 6]. TAE is mostly regarded as a second-line treatment after endoscopic haemostasis failure. The technical and clinical success rates of TAE in patients with GC bleeding are 85.0%–100% and 40%–72.5%, respectively [12–14]. The clinical success rate of TAE for tumour staining is high [12]. Considering the effectiveness of TAE for tumour staining and the difficulty of endoscopic haemostasis in large tumours, it may have been reasonable to perform TAE in our cases because delayed TAE after endoscopic haemostasis failure can also have a worse prognosis than TAE performed earlier after only diagnostic endoscopy [13]. We report that preoperative embolization is effective for subsequent elective surgery in patients with severe neoplasm bleeding [15]. According to the National Clinical Database, a nationwide surgery registration system in Japan, minimally invasive procedures are less frequent in emergency gastrectomies [5]. In the present cases, we successfully performed laparoscopic total gastrectomy with curative resection as an elective surgery.

## Conclusion

Despite our limited experience, and the need for future studies to establish the appropriate indications, preoperative TAE may serve as a bridge to curative and minimally invasive surgery, thereby reducing the need for emergency surgery, which is associated with high postoperative complication rates, in patients with potentially resectable GC. However, the benefits of this approach require further validation and, understanding of the importance of careful patient selection for successful treatment.
